# The death of Rasputin—A forensic evaluation

**DOI:** 10.1007/s12024-024-00793-9

**Published:** 2024-02-22

**Authors:** Roger W. Byard

**Affiliations:** Adelaide School of Biomedicine, The University of Adelaide, Level 2, Room N237, Helen Mayo North, Frome Road, Adelaide, 5005 SA Australia

**Keywords:** Rasputin, Death, Execution, Shooting, Poisoning, Drowning, Little Nevka River

## Abstract

Grigori Yefimovich Rasputin, a confidant of Tsar Nicholas and his wife, was murdered by Prince Yussupov and his co-conspirators in the cellar of the prince’s Moika Palace in St Petersburg, Russia, on the evening of December 30th, 1916 (December 17th in the Russian calendar). The narrative of his death is largely based on Prince Yussupov’s published memoirs and has Rasputin being poisoned with cyanide, shot, bludgeoned, and finally drowned. A review of the available forensic material, however, shows a photograph with a contact gunshot wound to Rasputin’s forehead. This would indicate that he was dead prior to being dropped into the Little Nevka River. His distaste for sweet foods and the absence of poison at autopsy would also suggest that the story of cyanide toxicity was fabricated. Yussupov’s description of Purishkevich firing at Rasputin from a distance as he ran across the courtyard in an attempt to escape would also not be consistent with the post mortem photograph. The simplest version of the events would be that Rasputin was executed by a contact gunshot wound to the forehead when he visited the Yussupov Palace. While it appears that the events of that fateful evening have been embellished, it is certainly not uncommon for perpetrators of homicides to provide histories that are later shown to be at odds with the truth. Re-evaluation of historic cases may provide compelling evidence for alternative interpretations to the popular historic record.

## Introduction

**Fig. 1:**
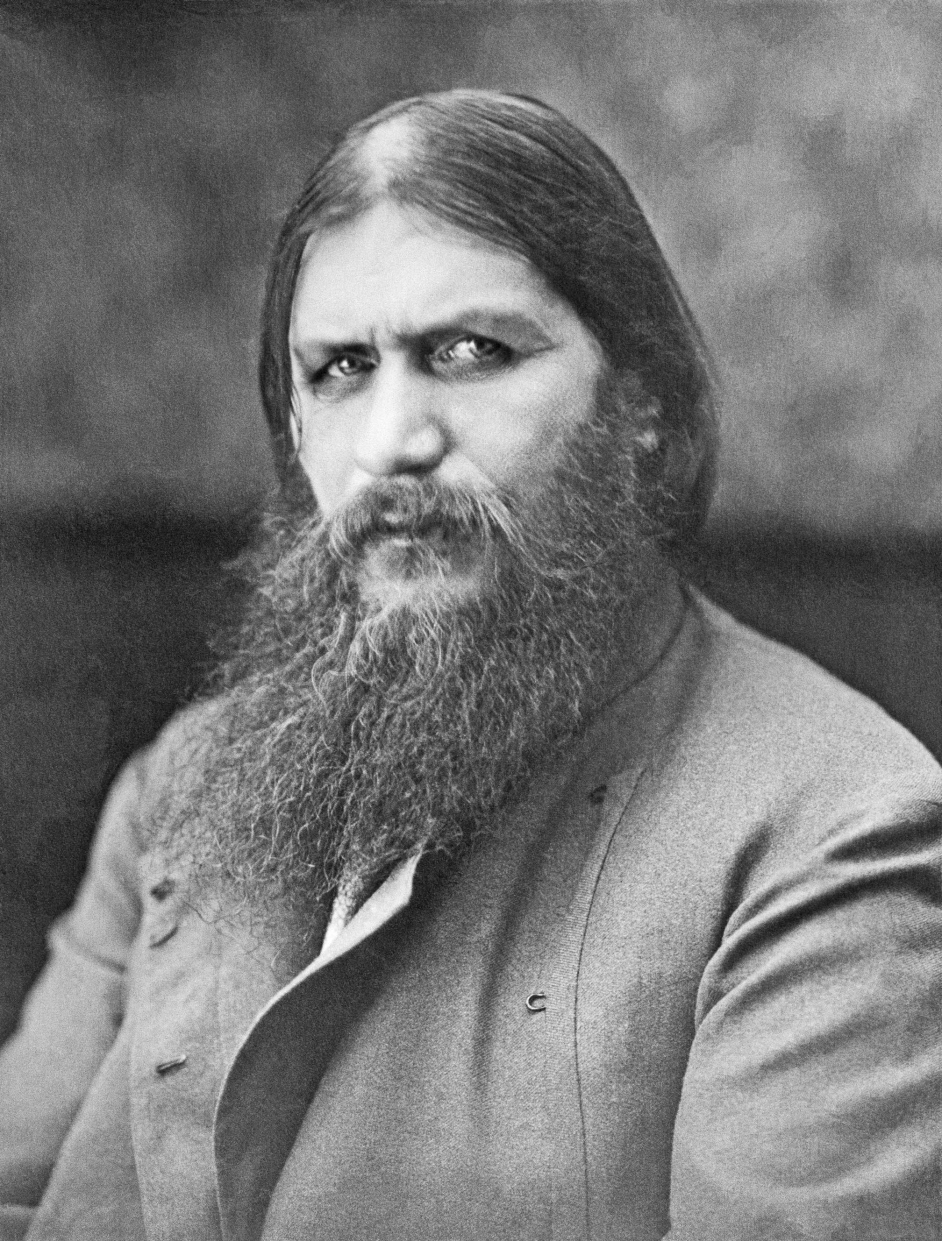
A photograph of Grigori Yefimovich Rasputin taken around 1910 (public domain)

Review of historical incidents from a contemporary forensic point of view can sometimes shed new light on particular occurrences or provide assessments of the most, or least, likely sequence of events [1, 2]. One of the enduring mysteries of the fall of the Romanov empire surrounds the death of the Tsarina’s confidant, the Siberian mystic Rasputin [3]. Grigori Yefimovich Rasputin (Fig. 1) was born as the son of a poor peasant in a small village in western Siberia on 22 January 1869, and was later destined to gain unprecedented access to the Romanov royal family prior to the disintegration of one of the world’s largest autocracies [3]. Despite his influence with the Tsarina Alexandra, it has been suggested that he was not necessarily a sinister puppet master manipulating political fortunes but rather more of a symptom of the developing troubles that were evolving around developing political and economic instability and social unrest [4]. He is still, however, remembered by such unflattering names as the ‘Holy Devil’ and the ‘Mad Monk’ [5, 6]. The following analysis concentrates on the events surrounding his death rather than on his apparent powers as a mystic healer, his alleged debauchery, and his possible political influence in the court of the Tsar.

## Early life

**Fig. 2:**
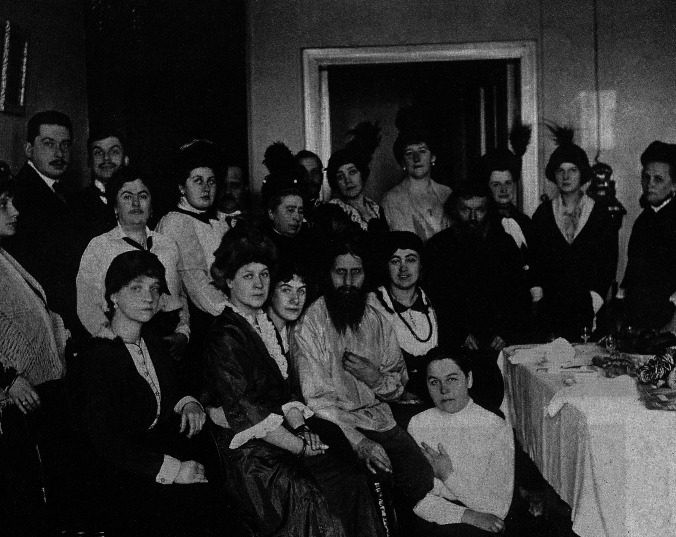
Rasputin surrounded by his admirers, most of whom were women (public domain)

Rasputin’s early life was characterised by purported mystical and healing powers, with a gift for prophecy. During a pilgrimage to a monastery in 1897, he experienced a religious conversion following which he was variously known as a *strannik* (pilgrim or wanderer), or as a monk, although he never held an official position in the Russian Orthodox Church [3]. In 1903, he first visited St Petersburg and, again, in 1905 where he met and was accepted by a number of senior figures in the church [7]. He was also befriended by two Montenegrin princesses who were interested in mysticism and who coincidentally were married to cousins of Tsar Nicholas II, leading to him becoming a recognised figure in Russian society [7] (Fig. 2).

## Time with the Romanovs

He first met the Romanovs when he was taken to Tsarskoe Selo by Grand Duchess Militsa on 1st November 1905 [7]. This was the beginning of the path to his murder 11 years later by a group of nobles led by Prince Felix Yussupov—the details of which have been strenuously debated over the past century.

The defining issues that led to Rasputin’s rise in power involved the Tsarevitch Alexis who suffered significant health issues from the ‘Royal disease’, haemophilia, that he had inherited through his mother from Queen Victoria [2, 8]. Next was the desperate need of his parents for therapies that might work.

**Fig. 3:**
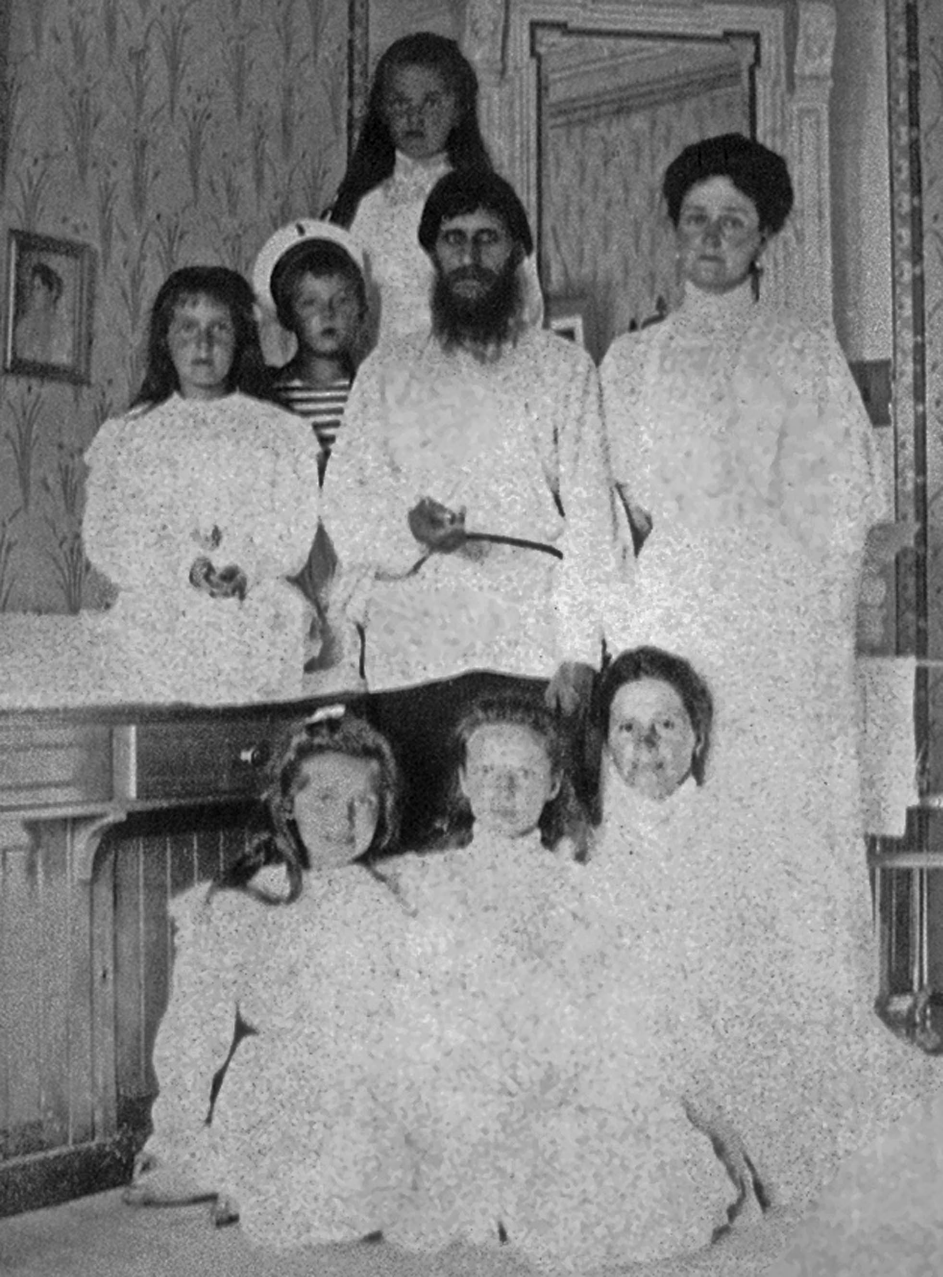
A photograph of Rasputin with the Tsarina Alexandra and her children (public domain)

**Fig. 4:**
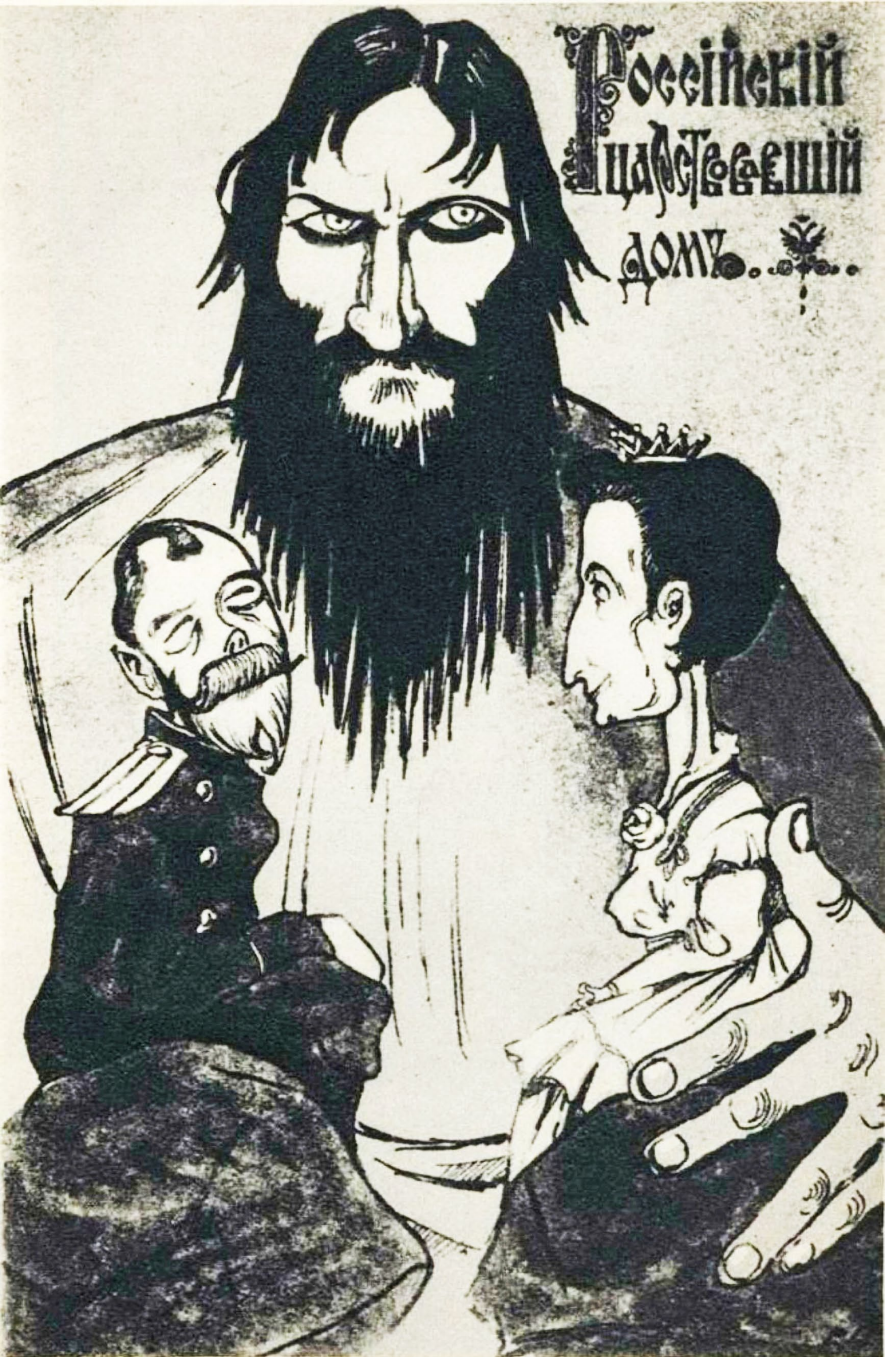
A poster showing a caricature of the evil Rasputin holding a simpering Nicholas and a controlling Alexandra clearly captured anti-Rasputin sentiments (public domain)

Rasputin was observed to have a settling effect on the young Tsarevitch, and it has been suggested that this may have been due to his ability to calm the Tsarina. It has also been proposed that his recommendations to withhold medical treatments may have resulted in the discontinuance of aspirin [9–11] which of course would have exacerbated bleeding in young Alexis. His powers as a healer were firmly believed in by Alexandra, and as a result, he was a frequent visitor to the Romanov family (Fig. 3). He was, however, not without significant enemies who were concerned about his apparent political influence with the Tsar, who was not the most skilled or astute of rulers [7]. Anti-Rasputin rumours, including that he was the lover of the Tsarina, and posters depicting his alleged power over the Romanovs proliferated (Fig. 4).

## The end

**Fig. 5:**
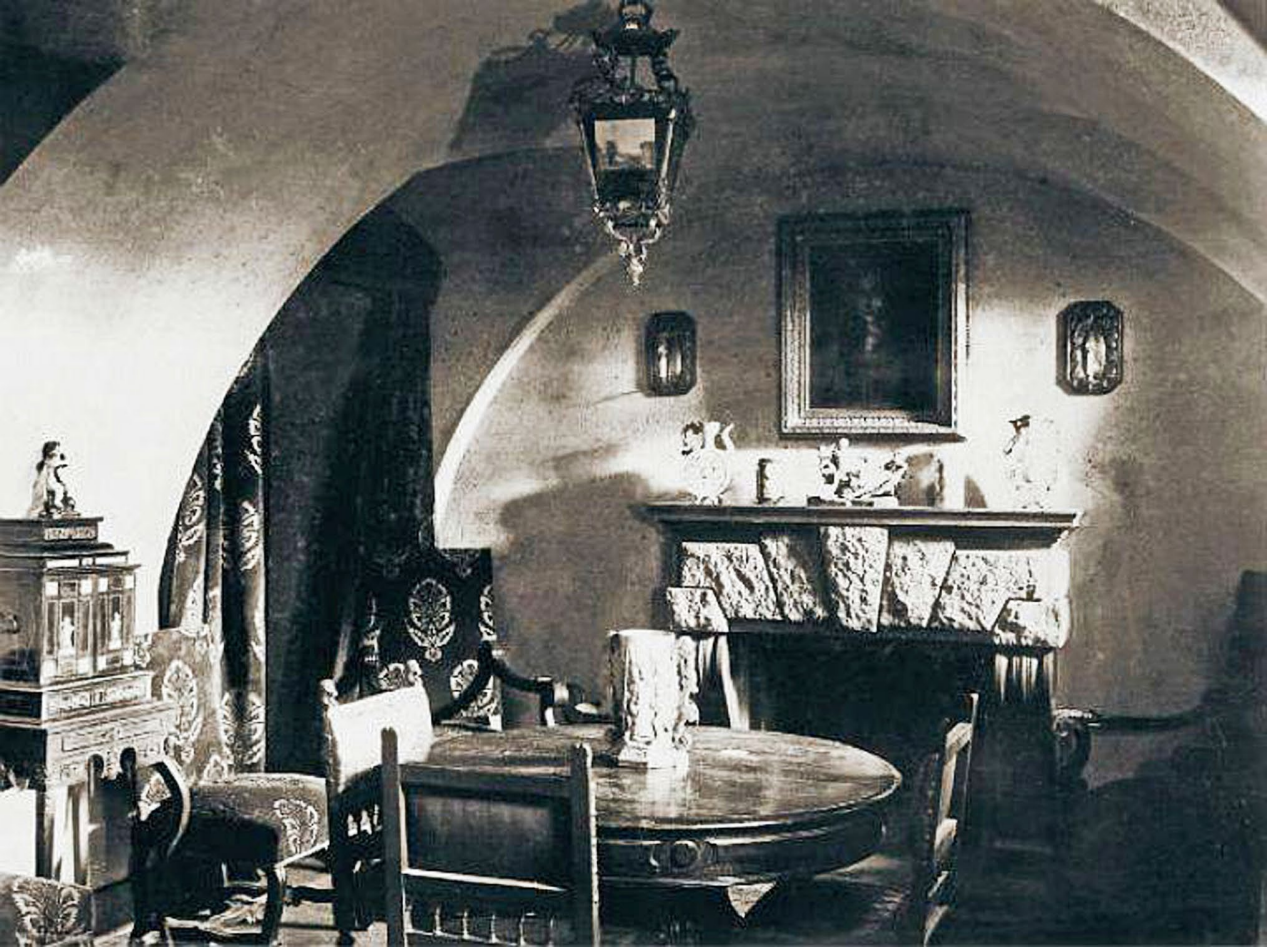
The basement room of Prince Yussupov’s Moika Palace where Rasputin was most likely executed (public domain)

**Fig. 6:**
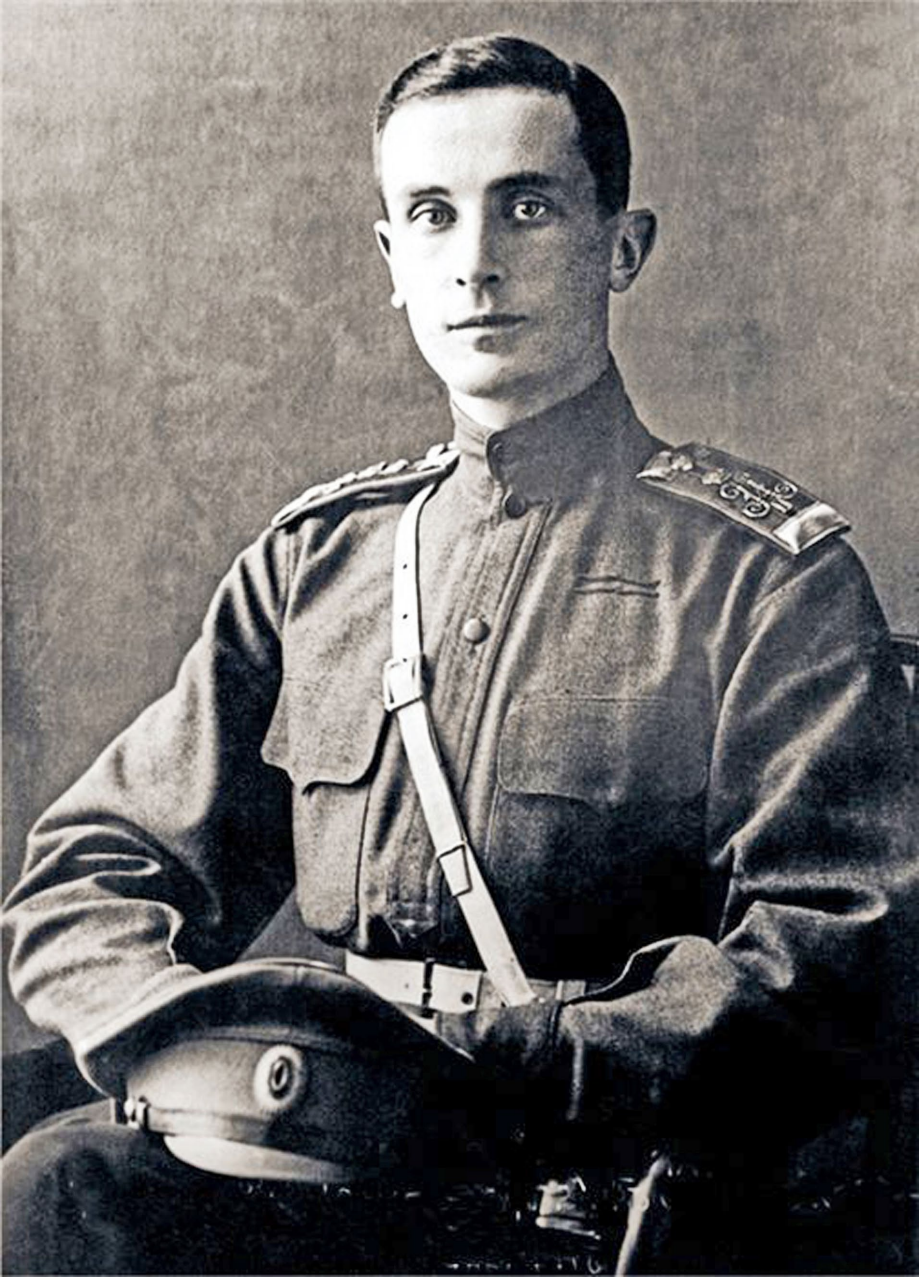
Prince Felix Yussupov in 1914 (public domain)

The details of Rasputin’s death have become the substance of legends over the years, much of which is based on Yussupov’s memoirs, published in 1928 [11]. What is clearly known is that Rasputin accepted an invitation to attend a social function in the cellar of Prince Yussupov’s Moika Palace on the evening of December 30th, 1916 (Fig. 5). Yussupov (Fig. 6) was married to Princess Irina, a niece of the Tsar, and was the sole heir to the largest fortune in Russia. Despite having previously socialised with him, Yussupov had decided that Rasputin represented a danger to the monarchy and therefore had to be eliminated [7]. One of the five conspirators, Dr Lazovert, allegedly laced both cakes and wine with cyanide which were given to Rasputin.

According to Yussupov, Rasputin was unaffected by the poison, and so he had to shoot him in the back/chest with a revolver. Although declared dead by Dr Lazovert, Rasputin later arose and attacked Yussupov chasing him into the courtyard where he was shot twice by Purishkevich (in the shoulder and head) [7]. The body was then brought back into the house where it is alleged that Yussupov battered it with a club before castrating it [3, 6]. The remains were then wrapped in a curtain and dropped from the Petrovsky Bridge into the Little Nevka River. Thus, the belief was that ‘Gregory Rasputin, his bloodstream filled with poison, his body punctured by bullets, had died of drowning’ [7], plus or minus the additive effects of blunt force trauma and possible castration [3].

On the instructions of the Tsarina, Rasputin was buried in the grounds of Tsarskoe Selo in January 2017, only to disinterred and burnt in March under orders from the new government [3]. Rasputin’s alleged letter to Nicholas in December 1916 was believed to be prophetic in that he asserted that if he was to be killed by *boyars (*nobles), then ‘none of your children or relations will remain alive for more than two years. They will be killed by the Russian people’ [7]. In keeping with this prediction, Nicholas, his wife, and five children were all executed by the Bolsheviks just 19 months later in July 1918 [2]. It has been suggested that the letter was in fact a later forgery that merely added to the mythology surrounding Rasputin [12].

## Forensic clarifications

While modern review of historical records can certainly clarify events from the past [1], the paucity of information can often make any conclusions merely conjectural [13]. In the case of Rasputin, the lack of availability of the original autopsy report complicates matters, although it must be recognised that even if historical official autopsy reports are found, they may not always accurately reflect the number and type of injuries. For example, although police statements regarding the death of the nineteenth-century Australian outlaw (‘bushranger’), Ben Hall, describe multiple gunshot wounds, the official autopsy report documented far fewer [14]. In the case of Rasputin’s death, there are a number of discrepancies between the police report and the accounts given by Yussupov and Purishkevich [6].

**Fig. 7:**
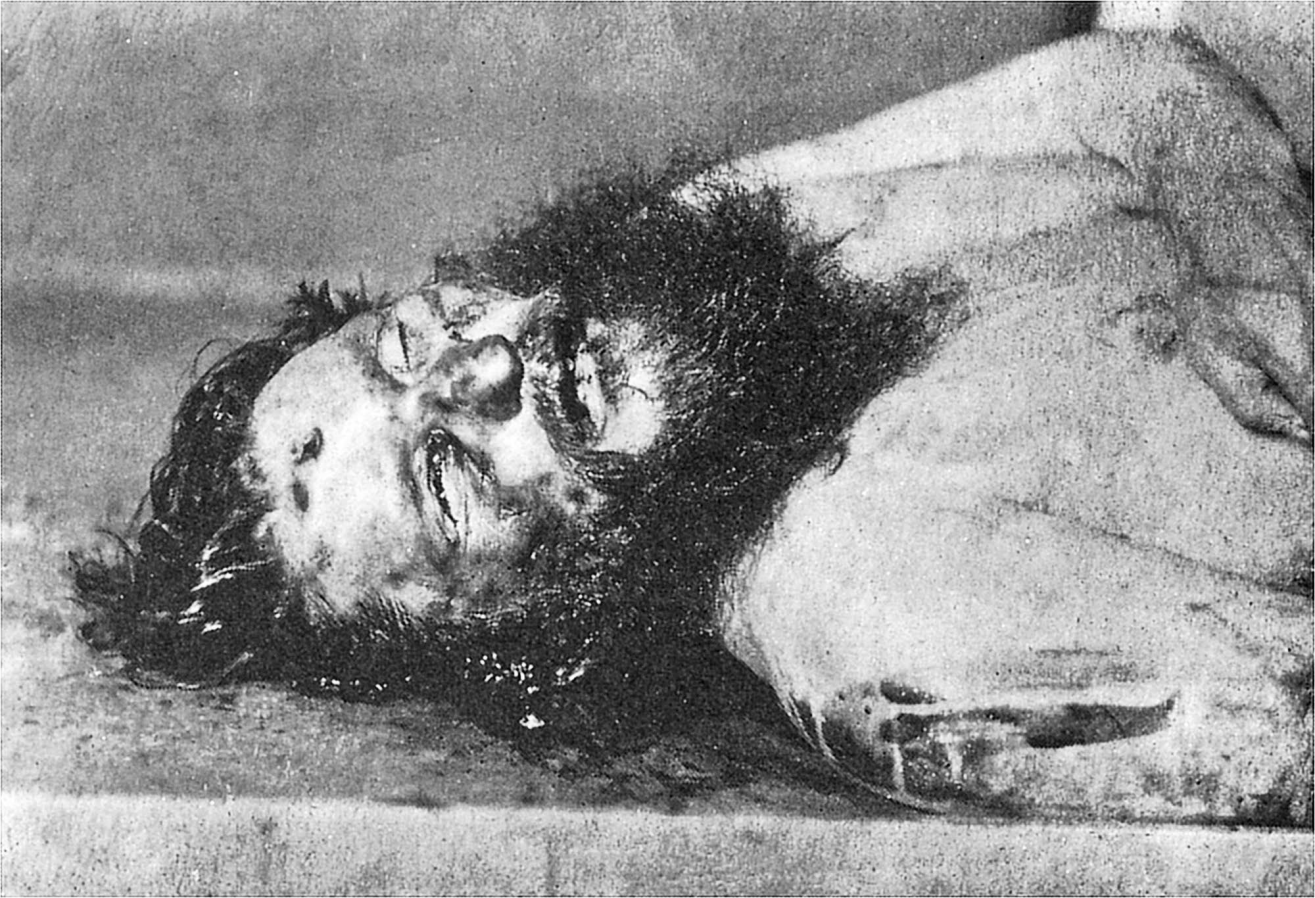
The most compelling evidence for the circumstances of Rasputin’s death is this post mortem photograph which shows a contact wound to the mid forehead with a muzzle imprint abrasion around the lower and right margins and no stippling or soot soiling (public domain)

A review of the original autopsy findings was conducted by Dr Zharov in 1993 [12]. This revealed that there were two bullet wounds to the liver and kidney, respectively, which were considered to have been inevitably lethal. Injuries were also found which included avulsion of the right eye (not clearly shown in the post mortem photograph of the body; Fig. 7), partial detachment of the right ear, crushing of the genitals (which were present), wounding of the neck with a blunt object, injuries to the face and body ‘inflicted by some flexible but blunt object’, and a sharp force wound to the left side of the back. It was concluded that the injuries to the head were caused by ‘a succession of blows inflicted by heavy blunt objects’ and that these could not have been caused ‘by the body hitting the pylon of the bridge’ [12]. These observations are of interest as it is known that Rasputin’s body was thrown off a bridge into an ice-filled river and retrieved using grappling hooks [10], all of which could cause or lead to significant injuries to the body. It is unclear given this history whether ante- from post-mortem injuries could be differentiated with such confidence.

A post mortem photograph of Rasputin was apparently discovered by police 2 months after the murder [12]. If this photograph is accepted as that of Rasputin, there is an obvious contact gunshot wound to the mid forehead (Fig. 7). This would have been immediately incapacitating and undoubtedly lethal and must have been inflicted prior to the body being dumped into the Little Nevka River. For this reason, it is not necessary to discuss whether drowning had occurred or not. As well, the observation in the autopsy report of an absence of fluid in the airways as an indicator that drowning had not occurred is also unnecessary, but incorrect, as it is now recognised that drowning may occur without any diagnostic pathological markers [15].

The contact wound depicted in Fig. 7 has a muzzle imprint abrasion visible around the lower and right margins and no stippling. The range does not fit with Yussupov’s story that Purishkevich had been firing from a distance at Rasputin as he ran across the courtyard in an attempt to escape [7]. Thus, both the range and the position of the gunshot wound in the central forehead do not fit with Yussupov’s description. By definition, a fleeing victim is not running towards his or her assailant and would logically be expected to sustain gunshot wounds to the back of the head and not the forehead. Professor Kossorotov in his original autopsy report actually documented that a gun had been ‘pressed to his forehead’ [12]. Although it has been asserted that this image of a single gunshot wound implicates a British Secret Service Officer in the shooting [12, 16], it in no way proves who did the actual shooting even if the weapon used was one carried by officers of the British Secret Service. The reasons for a suggested British involvement in the murder were to do with Rasputin’s opposition to the war with Germany [17].

The issue of Rasputin’s failure to succumb to food and drink that had been poisoned with cyanide could be easily explained by the doctor’s subsequent statement that for some reason, he had at the last minute baulked at lacing the meal with poison [6, 10]. The failure to find cyanide noted by Professor Kossorotov at post mortem—‘the examination reveals no trace of poison’ [12]—would be supportive of this and would further demystify the terminal events [9]. Another inconsistency in the conspirators’ story is that it was known that Rasputin did not eat sweet foods as he feared that this would impair his special powers [18].

## Conclusions

The lethal gunshot wound to the forehead means that Rasputin was dead before being dropped into the river. Whether the shot was in the form of an execution, or as a ‘coup de gras’ following the other gunshot injuries, is uncertain, although it would seem unnecessary to shoot him in the chest and back once the head injury had been inflicted. The need to use gun(s) and absence of poison at autopsy also suggests that the story of lacing food with cyanide was not correct.

Harris states that ‘The autopsy reports do not mention poison or drowning but instead conclude that he was shot in the head at close range’ [11]. Thus, the simplest version of the events would be that when Rasputin visited the Yussupov Palace on that night in December 1916, he was fatally shot. Of note, no one was ever arrested or charged with the murder.

While the reasons for Yussupov’s embellishments remain unclear, it has been suggested that the intent was to transform ‘the murder into an epic struggle of good versus evil’ essentially to help sales of his book [11]. It is also certainly not uncommon for perpetrators of homicides to provide histories that are later shown to be substantially different to the truth. Contemporary evaluation of even the most rudimentary surviving evidence, such as a single post mortem photograph, may therefore enable the crafting of reasonable alternative explanations.

## Data Availability

There is no original data as it is a lesson from the museum.
